# Vision Inspection Method for the Quality Assessment of Paint Coatings on Glassware

**DOI:** 10.3390/ma17184566

**Published:** 2024-09-17

**Authors:** Damian Dubis, Andrzej Chochół, Izabela Betlej, Piotr Boruszewski, Piotr Borysiuk

**Affiliations:** 1 Institute of Technology, State University of Applied Sciences in Krosno, Rynek 1, 38-400 Krosno, Poland; damian.dubis@pans.krosno.pl; 2 Department of Metrology and Instrumental Analysis, Cracow University of Economics, Rakowicka 27 st., 31-510 Kraków, Poland; chochola@uek.krakow.pl; 3 Institute of Wood Sciences and Furniture, Warsaw University of Life Sciences—SGGW, 159 Nowoursynowska St., 02-776 Warsaw, Poland; piotr_boruszewski@sggw.edu.pl

**Keywords:** quality inspection, coating resistance, non-contact measurement, computer vision, glassware

## Abstract

Image analysis is becoming increasingly popular in many industries. Its use is perfect for, among other things, assessing the quality of products on or off the production line. Highly automated, high-performance systems can be used for this purpose. However, there are situations in which automated vision systems cannot be used on the production line due to the specific nature of the process. One such situation is testing the resistance of paint applied to glass when washing in automatic dishwashers. It is carried out outside the production line, and typical production vision systems are not used here. An attempt was made to develop a cheap and easy-to-implement research method enabling quantitative measurement of paint loss on glass when testing the coating’s resistance to automatic washing. For this purpose, analysis of images taken during the study was carried out. The developed method is based on taking a series of photos of the tested object between each stage of the wash resistance test. The obtained photographic material is then analyzed by measuring the size of paint losses expressed in the number of pixels. Then, the percentage of paint loss is calculated. This method is cheap to implement and highly accurate. Statistical analysis of the results confirmed the method’s accuracy at 98%.

## 1. Introduction

Computer vision research and applications have their origins in the 1960s. In the past decades, machine learning techniques have contributed to noteworthy progress in vision systems. To achieve this, vision systems can be comprised of different subtasks, which are grouped under three categories: low-level vision, intermediate-level vision, and high-level vision. Low-level vision tasks comprise operations such as image acquisition and pre-processing. Intermediate-level tasks pertain to segmentation, symbolic representation, classification, and recognition. High-level vision tasks concern conceptual understanding of information acquired from lower-level vision modules. Currently, computer vision is a well-established field of research with a focus on intermediate-level applications, such as the automation of tasks in production lines in industrial settings [1].

Automated vision inspection systems are widely used in various industrial areas, e.g., in checking the quality of wooden floors [2], textiles [3], cigarette packs [4], metal elements [5,6], and 3D prints [7]. Food quality evaluation, for example, beef [8], cheese [9], fruit, [10] tea [11], blueberries [12], and wafers [13], can also be performed with this method. Also, such systems are commonly used for dimension [14] and volume [15] measurements of various objects, curved surface inspection [16], and assembly process control [17,18]. Regarding glass products, researchers have developed automated optical inspection devices using advanced image processing methods to detect the shape and surface of glass products (such as lenses) and analyze their optical properties [19]. Other researchers have focused on automatic defect detection on optical [20], flat [21], or bioactive glass [22].

Most of the related studies focus on the deformation detection of optical glasses. Only some papers have applied optical inspection systems to assess tableware glass quality.

Dishwashers are becoming increasingly popular pieces of household equipment. This fact creates new challenges for glass manufacturers, as it becomes necessary to use paints for decorating that are highly resistant to mechanical washing. During the production process, it is necessary to test and compare different types of paints applied to glass in terms of their resistance. The paint must be resistant to washing. Paint resistance depends on several factors: the quality of the paint itself, glass surface cleanliness (lack of dust), and surface preparation method (for example, by flame burning). In industrial practice, the results of glass-washing resistance tests prove not only the quality of the paint itself but also the quality of the entire application process. Lack of sufficient resistance to washing in the dishwasher can be a reason for consumer dissatisfaction. In recent years, significant progress has been made in improving the resistance of decorations to washing. However, only some types of paint are sufficiently resistant to washing [23,24,25]. Moreover, so far, there is no objective test of the adhesion of paint to the glass surface. A popular and sometimes recommended method is the “scotch tape” method—which involves sticking adhesive tape to the decoration applied to the glass and then vigorously tearing it off [26]. The absence of decoration defects indicates a positive test result, and traces of paint on the tape, a negative result. However, the results obtained in such a test are unreliable because they depend on many factors: the force with which the tape was glued to the glass, the type of adhesive applied to the tape, and the strength of the person peeling it off the surface. Another test method involves rubbing off a layer of glass paint with sandpaper of a certain grit, pressed against the surface under constant pressure [26]. However, this study does not even remotely reflect the conditions affecting glass in automatic dishwashers, so it is unreliable in assessing the product’s quality.

Industry standards describe methods for assessing the resistance of glass to the mechanical washing process, but the criteria for this assessment are biased. They are based because of terms such as: “no visible changes,” “first noticeable change,” and “clearly visible changes.” These types of assessment are qualitative, not quantitative. They are based on a visual estimate of the extent of paint damage [27].

This paper aims to describe a new method for the quality assessment of paint coatings on glassware. The model it is based on is quite well-known in the literature [10]. This model contains three stages: image acquisition, measurement, and decision. However, the generic model must be adapted to each new application. It has, therefore, been transformed into a new method of testing the resistance of paint coatings to the mechanical washing process.

An original research method was developed that meets the following assumptions:

- The test should enable quantitative measurement of the amount of paint loss and lead to obtaining a precise and objective result;
- The measurement method should be as simple as possible and be able to be used without the need for highly specialized tools or software;
- The possibility of implementing the developed measurement method in industrial practice should be as comprehensive as possible.

The innovative aspects of this study are as follows:

- The developed method is quantitative in nature, so it is much more precise than existing qualitative methods;
- The developed method improves the detection of defects, even small ones, because some of them cannot be detected by the naked eye;
- Vision inspection systems are widely and successfully used in various industries, and now they can be implemented for another new application in the glass industry;
- High accuracy makes the results very reliable, and the more reliable the results, the easier it is to prove the quality of the product to the customer.

## 2. Materials and Methods

The Canon PowerShot digital camera was used as a research tool to obtain photographs. Adobe Photoshop CS2 version 9 software was used to analyze the photos. For method testing, glasses with a capacity of 50 mL decorated with thermoplastic paint were used. Thermoplastic paints are commonly used in the glass industry. Typical components of these paints are pigments, resins, and solvents. At temperatures below 60 °C, they are in solid form, but after heating to temperatures above 60 °C the ink acquires a semi-liquid form, which enables printing using the direct screen printing method. The paint itself adheres to the surface of the glass. After the decoration is applied, the products are heated in the oven. This treatment makes the decoration permanent and glossy. Glasses were decorated with small squares (1 mm ± 0.1 mm square size) and circles (1 mm ± 0.1 mm circle diameter) around the cup’s circumference. The diameter of the glasses was 45 mm ± 1 mm. The height was 150 mm ± 1 mm. The decoration was made using the direct screen printing method. This method ensures a high repeatability of decoration throughout the entire batch of products, which is statistically confirmed in the Section 3.

**Figure 1:**
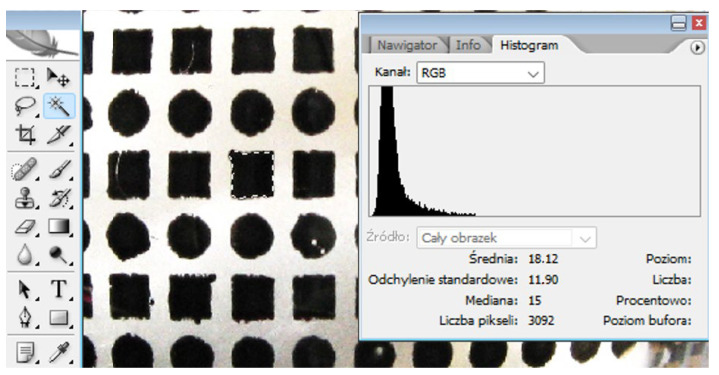
A view of the software interface (Adobe Photoshop CS2 version 9) and a fragment of a photograph were taken. A wand tool (highlighted in blue) was used to mark the object of interest (object of interest is marked by the dashed line). Histogram tools were used to measure the number of pixels. After marking an object of interest with a wand tool a histogram tool shows a number of pixels. Meaning of words in the figure: średnia—mean value; odchylenie standardowe—standard deviation; mediana—median; liczba pixeli—number of pixels; poziom—level; liczba—number; procentowo—percentage; poziom bufora—buffer level.

The principle of the developed method is based on determining the percentage loss of mechanically cleaned decorations through computer analysis of graphic files showing the condition of the decorations at individual stages of the experiment. This analysis involves separating the areas covered with paint in each photo and measuring the number of pixels in a given area using graphic photo processing software (Figure 1).

**Figure 2:**
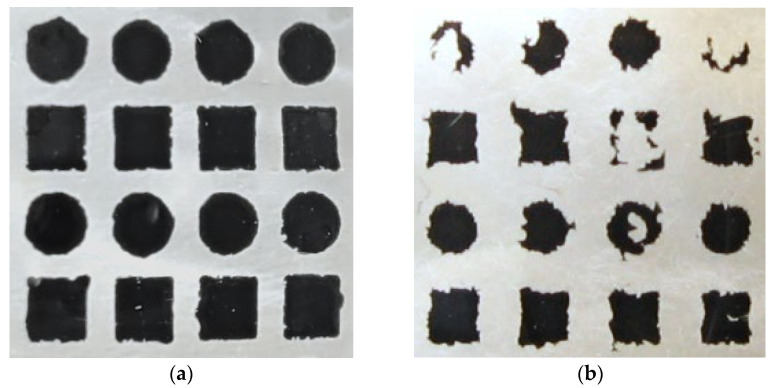
An enlarged fragment of product decoration: (**a**) undamaged; (**b**) damaged.

After subsequent washing procedures, the surface covered with paint decreases, thus reducing the number of pixels in the photo. An example is shown in Figure 2, which shows the appearance of the same decoration fragment before and after six washing cycles. The decoration was made with organic paint. During washing, the decoration is damaged. Only fragments of it remain on the glass. These fragments have irregular shapes.

In the experiment, photographs were taken in the following conditions:

- Camera mode: full manual settings;
- Exposure time: 1/13 s;
- Aperture value: f/2.0;
- Photo resolution: 2272 x 1704 pixels;
- Lighting: two LED bulbs 600 lm/W;
- Value of the color rendering index of bulbs: 85;
- Zoom: no zoom;
- Distance between the camera and the tested product: 15 cm.

**Figure 3:**
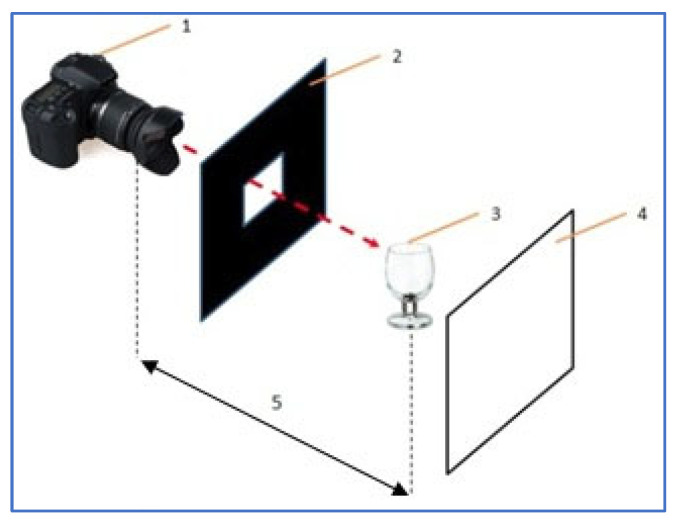
Schematic diagram of developed vision system; 1—camera; 2—black background; 3—photographed object with white filling inside; 4—white background; 5—constant distance between the camera and the tested object. A dashed arrow indicates that the camera should take a picture of the glass through the hole in the black background.

It is essential that the background of the photographed object is of uniform color to eliminate the possibility of reflections of surrounding elements in the photographed glass or paint. The experiment used a system of two shutters, black and white. Moreover, the product’s interior should be filled with white material so that only the front wall of the product is visible when photographing. The workstation diagram is shown in Figure 3.

The distance between the tested product and the camera is of crucial importance. Since the method is based on determining the percentage of paint loss (relative units) and not the area of loss (absolute units), it is crucial that the distance from which the photos are taken is always the same (e.g., 15 cm) and is maintained throughout every stage of the experiment. Then, the decreasing number of pixels in the photos throughout the experiment will be caused by the actual loss of paint, not by the change in the distance between the camera and the tested object.

**Figure 4:**
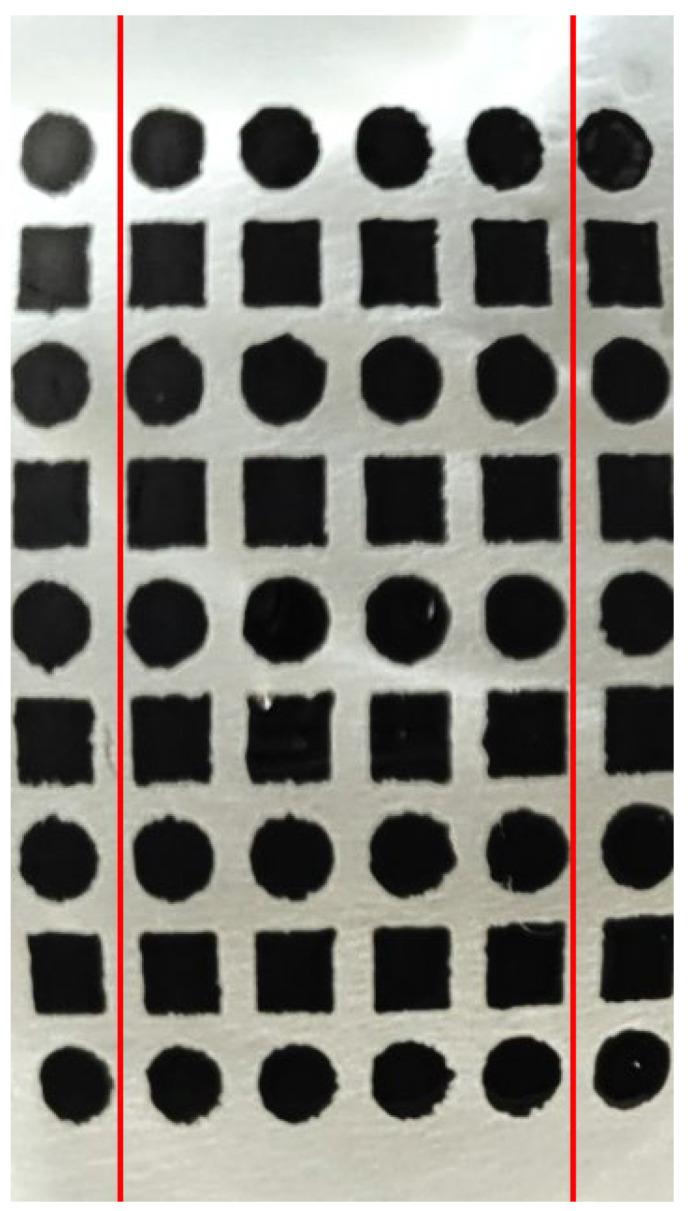
An example of a tested section isolated on one of the tested samples. This section is marked with two red lines.

Another critical factor affecting the quality of the obtained photographic material is the decoration fragmentation method. If the decoration is placed around the glass, showing it in only one photo is impossible. It often happens with round utensils, for example, cups, mugs, glasses, and tumblers. In that case, taking several photos from each side of the tested product is necessary. So, the entire decoration should be divided into several sections. Every section should be separately photographed (Figure 4). Also, round glasses produce light reflections on the edges when being photographed. To avoid this affecting the results, a region of interest (ROI) must be extracted from each sample. Therefore, if the decoration is applied around the entire circumference of the tested product, it should be divided into 15 equal sections (with angles of about 25°), and each section should be photographed separately. In the tested samples, the width of a section corresponds to five columns made of individual elements. That prevents positioning errors and light reflection at the edges, affecting the results. If necessary, the sections should be marked with a permanent waterproof marker directly on the surface of the glass. This prevents the sections from being mistaken with one another between washing cycles.

In order to determine the necessary size of the research sample to ensure the reliability of the results, an experiment was carried out in which a preliminary statistical analysis was made of the number of pixels that make up individual decoration elements in the photographs taken. Glasses decorated with a pattern consisting of squares and circles arranged alternately on the glass were used for the analysis. This analysis included the following:

- Determining the values of basic statistical measures of the concentration of results (arithmetic mean), the dispersion of results (minimum value, maximum value, standard deviation, coefficient of variation), and the shape of the distribution (skewness coefficient).
- Determining the necessary number of observations (sample size)—N_min_. For this purpose, Formula (1) was used:

N_min_ ≥ [t^2^_1−α; n−1_ × υ^2^]: d^2^ (1)

where

t^2^_1−α; n−1_—value of the Student variable “t” for n − 1 degrees of freedom and the assumed probability of meeting the inequality |$\bar{x}$ − μ_x_| ≤ d; probability was assumed = 0.95;
υ—coefficient of variation;
d—relative accuracy of value estimation μ_x_ [d = (Δx/μ_x_) × 100]; μ_x_ = 100%. The calculations were made assuming that d = 2%.

The result (N_min_) refers to what the minimum sample size should be to guarantee achieving the assumed accuracy of estimating the average value (μ_x_) in the general population using the average value ($\bar{x}$) from the sample [28].

The analysis was carried out in two ways, trying to indicate the more accurate method among the two possible ones:

- Photographing individual decoration elements (selected squares or circles) and, based on the measurement results of these elements, calculating the loss of this decoration;
- Photographing several element “sections” of decorations and, based on the results of section measurements, calculating the loss of decoration.

A total of 720 photographs were processed at this stage of the research. The preliminary analysis was prepared based on the following:

- Photographs of 120 randomly selected elements arranged on four samples, thirty on each of them;
- Photographs of the entire decoration on four samples. The decoration was divided into fifteen sections, each of which was photographed ten times.

## 3. Results

### 3.1. Method Development

Initially, it should be considered whether it is necessary to photograph all the tested products from all sides, as it consumes much time. The method should provide a reliable result with as few photos as possible. That is why statistical analysis was performed. The purpose of this analysis was to determine the minimum number of samples, as well as the images taken, to ensure that the results were sufficiently accurate.

Typical decoration glass for dishwasher use is made by direct screen printing with organic or ceramic paint. There are also many different methods (e.g., hand painting) and paint types (e.g., gold, and platinum varnish), but they are mostly not dishwasher suitable. So, for testing purposes, using samples made by direct screen printing with organic paint is reasonable. This method ensures high repeatability of decoration throughout the entire batch of products, which has been statistically confirmed.

The tested decoration consists of squares and circles. The number of pixels for randomly selected single decoration elements on individual samples ranged from over 3200 to approximately 3700 (squares) and over 2650 to 3150 (circles). The dispersion of the obtained results was small—extreme values ranged from approximately 2350 to approximately 4200. This is also indicated by the values of the coefficient of variation, which ranged from 5.9% to 7.4%. The values of the skewness coefficient were shallow (close to 0), indicating that the distribution of the results was symmetric.

The Nmin values of the necessary number of decoration elements ranged from 6.0 to 9.9. Therefore, it can be said that measuring ten randomly selected squares and ten circles (on each object) guarantees achieving the assumed accuracy of estimating the average value in the general population using the average value from the sample—with an error of at most 2%. Table 1 contains the results.

The N_min_ values of the necessary number of glasses are 8.8 and 8.2. Therefore, performing measurements on at least nine randomly selected samples guarantees the achievement of the assumed accuracy of estimating the average value in the general population using the average value from the sample with an error of at most 2%. Table 2 contains the results.

The sum of pixels for all decoration elements located in individual sections of the randomly selected samples ranged, on average, from 101.144 to approximately 115.143. The results’ dispersion was very low, and the values of the coefficient of variation did not exceed 6%. The skewness coefficient values were shallow, proving that the distributions of results are symmetric or slightly asymmetric.

N_min_ values ranged from 1.1 to 6.8. Therefore, selecting seven sections for measurements on each object guarantees achieving the assumed accuracy of estimating the average value in the general population using the average value from the sample with an error of at most 2%. A detailed summary of the results is provided in Table 3.

The number of pixels for all decoration elements on the randomly selected samples ranged, on average, from 101.147 to approximately 114.198. The dispersion of the results obtained was very low. This is also indicated by the coefficient of variation values, which did not exceed 6%.

N_min_ values ranged from 3.1 to 10.1. Selecting ten samples for measurements guarantees achieving the assumed accuracy of estimating the average value in the general population using the average value from the sample—with an error of at most 2%. A detailed summary of the results is included in Table 4.

So, to estimate the size of the loss using an average value from the sample, with an assumed accuracy of 98%, it is necessary to do one of the following:

- Take one photograph of each of the twenty randomly selected elements on each of the nine samples (useful, for example, if the decoration on the glass consists of single elements; if there are fewer than twenty elements, then all of them should be photographed), or;
- Take one photograph of each of the seven randomly selected sections of the decoration on each of the ten samples (useful, for example, if the whole surface of the glass is covered with paint; therefore, there are no single elements to choose from).

For the “single elements” choice, all the calculations should be performed according to Equations (2)–(6) below:

(2) $${PRP}_{k}=\frac{\bar{pixk}}{\bar{pixb}}\times 100\%$$

(3) $$\bar{pixk}=\frac{\sum_{i=1}^{9}{pixk}_{i}}{9}$$

(4) $$\bar{pixb}=\frac{\sum_{i=1}^{9}{pixb}_{i}}{9}$$

(5) $$pixk=\sum_{i=1}^{20}{pix}_{i}$$

(6) $$pixb=\sum_{i=1}^{20}{pix}_{i}$$

where

*PRPk*—the percentage of remaining paint after *k* testing procedures;
$\bar{pixk}$—arithmetic mean value of the *pixk* parameter calculated for nine samples after k washing cycles;
$\bar{pixb}$—arithmetic mean value of the *pixb* parameter calculated for nine samples before washing;
*pixk*—total pixels in twenty elements of a single sample after *k* washing procedures;
*pixb*—total pixels in twenty elements of a single sample before washing;
*pix*—number of pixels in a single element of decoration.

For the “sections” choice all the calculations should be performed according to Equations (7)–(11) below:

(7) $${PRP}_{k}=\frac{\bar{pixk}}{\bar{pixb}}\times 100\%$$

(8) $$\bar{pixk}=\frac{\sum_{i=1}^{10}{pixk}_{i}}{10}$$

(9) $$\bar{pixb}=\frac{\sum_{i=1}^{10}{pixb}_{i}}{10}$$

(10) $$pixk=\sum_{i=1}^{7}{pix}_{i}$$

(11) $$pixb=\sum_{i=1}^{7}{pix}_{i}$$

where

*PRPk*—percentage of remaining paint after *k* testing procedures;
$\bar{pixk}$—arithmetic mean value of the *pixk* parameter calculated for 10 samples after *k* washing cycles;
$\bar{pixb}$—arithmetic mean value of the *pixb* parameter calculated for 10 samples before washing;
*pixk*—total pixels in seven sections of a single sample after *k* washing procedures;
*pixb*—total pixels in seven sections of a single sample before washing;
*pix*—number of pixels in a single section of decoration.

### 3.2. Method Testing

In industrial practice, paint quality tests can last from several dozen to several hundred washing cycles, depending on the paint resistance. The entire research program is as follows:

- Photograph the samples before washing to determine the initial number of pixels that make up the decoration being tested;
- Put the samples in the dishwasher and do the first wash cycle;
- After the washing cycle, take the samples from the dishwasher, place them in front of the camera, and take photos. Ensure that the exact distance is maintained between the sample and the camera each time the sample is placed in front of the camera. This prevents positioning errors from occurring;
- Then measure the number of pixels in the photos and calculate the average paint loss;
- Then put the samples in the dishwasher and perform another wash cycle;
- The procedure is repeated until all the paint is removed from the test area.

The developed method has been tested, to check whether it could be suitable for industrial purposes. For testing purposes a small-scale experiment was performed. The durability of the paint was determined using three different commercial detergents. Three batches of tested products were washed, each with a different detergent. The purpose of this was to check the developed method in practice. Another purpose was to check for any paint resistance differences between tested batches. For obtaining the results, the “single elements” variant of the developed method was used, with an accuracy of 98%. So the number of samples was 27 (9 for each detergent) and 20 randomly selected elements were photographed on every single sample. The results were calculated according Equations (2)–(6).

**Figure 5:**
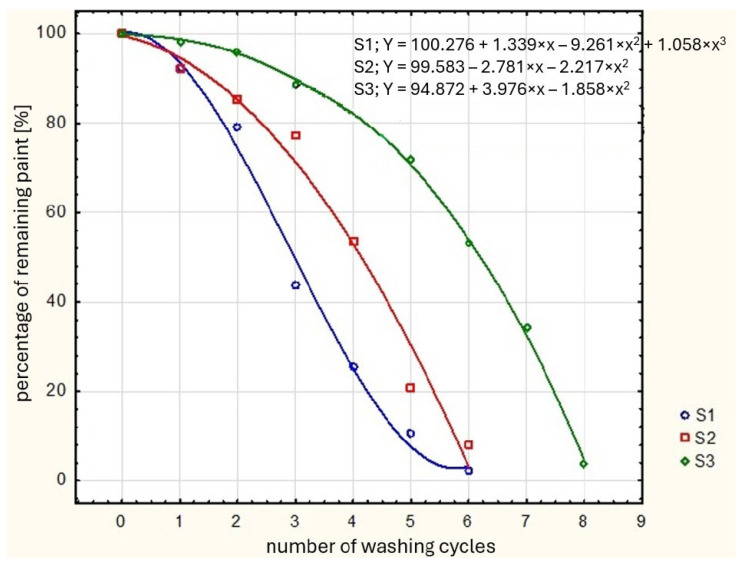
Example of a result obtained with the described research method. Detergent S1—blue line with blue circles; detergent S2—red line with red squares, detergent S3—green line with green diamonds.

The higher the percentage of remaining paint after washing, the higher the durability of the paint. Follow-up research on three products was conducted. The worst results were observed with detergent S1—very little paint remained on the glass after washing. This means that the detergent marked as S1 destroys decoration very quickly. The best results were obtained with detergent S3—the paint stayed on the product surface longer than with detergent S1 and S2. An example is shown in Figure 5. This research should be continued as it seems that the developed method would give exact and comparable results.

## 4. Discussion

Visual assessment, performed by even the best quality controller, is needed. This is because we cannot measure the size of paint loss by bare-eye assessment. The methods currently used are based on an estimated assessment of paint durability. Terms such as “small destruction of decoration” and “significant destruction of decoration” are used [27]. However, the area of damage needs to be precisely measured. Therefore, the results of such tests are only approximate and may be inaccurate. In this paper, a new, better method has been described, allowing us to estimate a paint’s durability with high accuracy. Washing resistance tests of glass products are usually conducted at two stages:

- At the prototyping stage, before production, to validate the prototype;
- At the end of the production process to make sure that the paint resistance meets the requirements from the prototyping phase.

The test is usually conducted in a separate room, such as a laboratory equipped with a dishwasher. These tests cannot be carried out directly on the production line. The dishwasher operates in a cyclic mode, which makes these tests time-consuming. It consists of several stages:

- Placing the tested samples in the dishwasher;
- Performing a fixed number of washing cycles;
- Removing the products from the dishwasher;
- Assessment of paint losses;
- Repeating steps (a) to (d) a fixed number of times;
- Final assessment of research results.

Each product should be placed in the dishwasher, removed after each washing cycle, and assessed for paint damage. After these steps, the products should be put back into the dishwasher, and another test cycle should be carried out. Sometimes, even more than a hundred cycles must be carried out. That is why the whole study is a lengthy one. A series of such tests can last as long as several weeks. Therefore, looking for ways to reduce the examination time is necessary. In practice, this can be done in two ways: reducing the time of the wash cycle or reducing the time for assessing product quality between wash cycles. Reducing the washing cycle time is usually not possible because the test conditions should be as close as possible to the conditions in which the customer will use the product. Therefore, it is necessary to wash the products using the washing cycle that the customer will use. Typical automatic dishwashers have wash cycles ranging from 30 min (suitable for lightly soiled dishes) to over 180 min (suitable for filthy dishes). The average washing cycle for medium-soiled dishes takes between 120 and 150 min. According to international standards, reference programs for research institutes can last almost 200 min [29]. The second solution, therefore, is to reduce the time needed to evaluate samples between wash cycles. The method proposed in this paper allows for the very rapid measurement of paint damage. In practice, the whole procedure requires taking several photos and measuring the size of selected decoration elements in pixels. The whole procedure takes several minutes.

In industrial practice, several hundred washing cycles are performed on the tested products, with a cyclical assessment of losses every five or ten cycles. In the case of very restrictive tests, the assessment is performed even after each washing cycle. The need to frequently assess, place, and remove products in the dishwasher makes it difficult to assess changes in paint loss on products. The fact that the tests performed are destructive makes the organoleptic assessment ineffective due to changes in the paint surface after each cycle.

It is also necessary to document the test results at individual stages, which necessitates taking photographs of the tested products at individual stages of testing. The above arguments support the implementation of vision methods for this type of assessment. However, vision systems adapted to mass production and installed on production lines cannot be used because a dishwasher cannot be installed there. The household dishwasher has a closed, chambered design and operates in cyclic, not continuous mode. The tests must, therefore, be performed in a separate laboratory and are lengthy. Unfortunately, this is a problem because tests must be performed simultaneously with ongoing production to quickly provide answers to whether the decoration is durable enough. There needs to be an improvement on current methods in this regard. Therefore, it is necessary to look for a way to reduce testing duration (and obtain more objective results). Since the washing cycle itself cannot be shortened (the manufacturer programs it and it must meet specific requirements), the method presented in this paper reduces the evaluation time between washing cycles.

Quantitative assessment of the loss of decoration involves determining the size of the loss on each tested object using the average value from the sample (with an assumed accuracy of 98%) and comparing it to the decoration’s average size before the washing process. This allows us to calculate the percentage loss of the tested decoration after each washing stage.

## 5. Conclusions

The proposed research method has high implementation potential because of the following:

- The test enables quantitative measurement of the amount of paint loss;
- It provides a precise and objective result;
- The method is straightforward and can be performed without the need for highly specialized tools or software;
- No lengthy staff training is required;
- The possibility of implementing the developed measurement method in industrial practice is wide.

The research method is highly accurate and easy to implement. It meets the assumptions described at the beginning of this work. The possibility of implementing the developed research method in industrial practice is significant. Each time, the researcher can choose between photographing a single element (which is better for decorations composed of many separate elements) or photographing whole sections (which is better for decorations covering a large area or even an entire product surface).

Similar results have been reported in the literature. For example, vision systems designed for food evaluation could do so with an error from slightly less than 5% [8] to approx. 2% [9]. However, color was measured there, while, in this work, it is the size. Another method, designed for the inspection and quality level determination of multifocal glasses, attains a 94% accuracy rate [19]. Some size measurement methods have an error rate of 2% [13] or even less [30]. For large parts, measurement method accuracy is approx. 92% or better [17]. The accuracy of the method presented in this paper is, therefore, comparable to the methods based on vision systems intended for other applications and described in the literature.

This method generates a lot of new research possibilities, including the following examples:

- Testing the resistance of decorated glassware against different cleaning detergents (this was briefly described in Section 3.2 of this paper as a way of testing the method);
- Testing the resistance of decorated glassware using different dishwasher types or cleaning programs;
- Testing the effectiveness of different cleaning programs for different types of soiling;
- Testing the effectiveness of different dishwasher types for different types of soiling;
- Testing the effectiveness of different detergents for different types of soiling.

The test method can be used by paint manufacturers, who can use it to test the paints they produce. It can also be used by glass manufacturers, who can test how a particular type of paint behaves in contact with a particular type of glass. Some manufacturers protect the glass surface from damage by applying various types of varnish. The effectiveness of such a varnish can also be tested by this method. Testing products to determine whether they meet customer requirements is also necessary. The more reliable the results, the easier it is to prove the quality of the product to the customer.

## Figures and Tables

**Table 1:** Values of basic statistical measures and the necessary number of observations for decoration elements.

	Sample No.	Mean	Min Value	Max Value	Std. Dev.	Variation Coefficient (%)	Skewness Coefficient	N_min_
square	1	3683.7	3298	4166	264.96	7.19	0.08	9.40
	2	3207.5	2862	3523	222.46	6.93	−0.01	8.74
	3	3227.5	2902	3547	190.38	5.89	0.07	6.34
	4	3256.6	3025	3668	227.00	6.97	0.32	8.84
circle	1	3148.9	2811	3485	190.78	6.05	−0.06	6.64
	2	2655.0	2371	2972	166.47	6.27	0.00	7.14
	3	2663.3	2347	2952	196.49	7.37	−0.05	9.90
	4	2651.6	2364	2970	177.67	6.70	−0.16	8.10

**Table 2:** Values of basic statistical measures and the necessary number of observations for samples.

	Mean	Std Dev.	Variation Coefficient (%)	N_min_
square	3343.8	157.48	4.71	8.8
circle	2779.7	126.18	4.54	8.2

**Table 3:** Values of basic statistical measures and the necessary number of observations (number of sections).

Sample	Photo	Mean	Min Value	Max Value	Std. Dev.	Variation Coefficient (%)	Skewness Coefficient	N_min_
1	1	104,642.4	97,141	113,785	4953.47	4.734	0.396	4.1
	2	104,612.0	96,646	113,469	4834.68	4.622	0.131	3.9
	3	104,626.5	97,723	112,533	4751.98	4.542	0.036	3.7
	4	104,872.3	98,934	112,381	4521.68	4.312	0.252	3.4
	5	105,190.9	99,329	113,020	4394.32	4.177	0.155	3.2
	6	105,081.4	98,083	113,015	4630.86	4.407	0.239	3.5
	7	105,343.5	97,205	114,486	5174.15	4.912	−0.104	4.4
	8	105,050.9	98,288	113,542	4990.95	4.751	−0.038	4.1
	9	104,906.9	96,934	113,447	5268.28	5.022	0.190	4.6
	10	104,951.1	98,022	113,314	4907.79	4.676	0.189	4.0
2	1	101,990.7	98,907	108,574	2764.55	2.711	0.917	1.3
	2	101,143.5	98,700	107,633	2504.49	2.476	1.336	1.1
	3	102,254.1	96,157	110,316	3426.66	3.351	0.635	2.0
	4	102,038.3	94,519	110,003	3643.20	3.570	0.162	2.3
	5	102,045.9	96,368	110,283	3411.47	3.343	0.775	2.0
	6	102,014.2	96,117	109,462	3647.17	3.575	0.426	2.3
	7	101,290.5	95,987	109,569	3492.32	3.448	0.730	2.1
	8	101,598.9	96,384	109,044	3691.33	3.633	0.576	2.4
	9	101,535.9	95,741	108,507	3659.72	3.604	0.540	2.4
	10	101,562.4	94,900	109,453	4235.65	4.170	0.661	3.2
3	1	103,024.7	97,236	111,825	3985.02	3.868	0.908	2.7
	2	101,886.3	95,130	112,017	4652.90	4.567	0.741	3.8
	3	102,284.5	96,221	110,258	3893.49	3.807	0.599	2.6
	4	102,346.3	95,997	109,118	3570.60	3.489	0.299	2.2
	5	102,279.4	96,908	109,267	3720.63	3.638	0.388	2.4
	6	102,667.7	98,888	109,048	3329.75	3.243	0.646	1.9
	7	101,681.6	97,812	108,923	3357.68	3.302	0.818	2.0
	8	101,378.4	95,367	109,515	3727.31	3.677	0.551	2.5
	9	101,806.2	95,987	109,997	4088.98	4.016	0.759	2.9
	10	101,448.1	95,262	109,148	3702.46	3.650	0.559	2.4
4	1	115,143.0	102,986	126,999	7008.34	6.087	0.011	6.8
	2	114,759.8	104,282	125,248	6840.60	5.961	0.000	6.5
	3	114,849.0	103,149	125,761	6910.14	6.017	−0.092	6.6
	4	114,180.3	102,898	124,102	6591.28	5.773	0.026	6.1
	5	114,127.8	105,136	123,999	6253.57	5.479	0.156	5.5
	6	113,632.7	105,296	123,919	5827.09	5.128	0.325	4.8
	7	113,833.3	103,996	124,954	6266.14	5.505	0.063	5.5
	8	113,745.1	103,259	126,530	6945.45	6.106	0.108	6.8
	9	113,683.3	102,286	123,915	7036.10	6.189	0.009	7.0
	10	114,025.8	103,314	123,721	6791.17	5.956	−0.074	6.5

**Table 4:** Values of basic statistical measures and the necessary number of samples.

Sample	Mean	Min Value	Max Value	Std. Dev.	Variation Coefficient (%)	N_min_
1	104,927.8	98,572.8	113,299.2	4757.217	4.53	6.3
2	101,747.4	96,574.4	109,284.2	3230.722	3.17	3.1
3	102,080.3	96,725.9	109,825.2	3582.919	3.50	3.7
4	114,198.0	104,168.5	124,208.1	6569.744	5.75	10.1

## Data Availability

The original contributions presented in the study are included in the article, further inquiries can be directed to the corresponding author/s.
